# Trends in tooth loss in relation to socio-economic status among Swedish women, aged 38 and 50 years: repeated cross-sectional surveys 1968-2004

**DOI:** 10.1186/1472-6831-13-63

**Published:** 2013-11-06

**Authors:** Anette Wennström, Margareta Ahlqwist, Ulrika Stenman, Cecilia Björkelund, Magnus Hakeberg

**Affiliations:** 1 Department of Behavioral and Community Dentistry, Institute of Odontology, The Sahlgrenska Academy, University of Gothenburg, Box 450, Gothenburg 405 30, Sweden; 2 Department of Oral and Maxillofacial Radiology, Institute of Odontology, The Sahlgrenska Academy, University of Gothenburg, Gothenburg, Sweden; 3 Department of Public Health and Community Medicine / Primary Health Care, The Sahlgrenska Academy, University of Gothenburg, Gothenburg, Sweden

**Keywords:** Tooth loss, Oral health, Socio-economic status, Women, Trends, Number of teeth

## Abstract

**Background:**

Oral diseases are a health problem worldwide. Differences in oral health status may vary with geographical locations, but also within the same country and between groups with different social backgrounds. The specific aims were to describe secular trends in oral health status regarding number of remaining teeth and also to describe differences in socio-economic status, among 38- and 50-year-old women, over a 36-year period.

**Methods:**

Cross-sectional health surveys were performed at four occasions; 1968/69 (n = 746), 1980/81 (n = 532), 1992/93 (n = 165) and 2004/05 (n = 500), including randomly selected women aged 38 and 50 years. The number of teeth was determined using panoramic radiographs and self-reported measures of marital status, social class, educational level, and income were recorded.

**Results:**

The mean number of teeth among women has increased significantly. The educational level has increased while fewer women are married/cohabiting over time. There has been a shift in the social group the women belong to, where proportionally more women were categorized in a higher social group in 2004/05 than in 1968/69. Moreover, there is a significant relationship between fewer teeth and a lower social group, and among the 50-year-old women, this was irrespective of examination year. However, multivariate analyses showed that the risk to be edentulous or not, or to have fewer remaining teeth was significantly higher for women of lower social group, or living alone, in all studies over the 36 year-period. This was independent of age group, even though the risk diminished over the study period.

**Conclusions:**

Cohort comparisons of women aged 38 and 50 years during 36 years showed that dental status improved, with (i) a decreasing prevalence of edentulism and, (ii) an increasing number of remaining teeth in dentate individuals over time. Differences due to social group and education were still present, with more remaining teeth in the women in the higher social group. A time trend analysis indicated that in the later examination years the individuals had fewer teeth lost, irrespective of age, marital status and, social group.

## Background

Oral diseases are a major public health problem, because of their high prevalence and incidence in the world [1]. In Europe and North America there are still substantial inequalities with regard to health and disease [2,3] and several studies reveal that there is a gradient between socio-economic status and oral disease; the lower the social status, the worse the individuals' oral health [3-6]. Material welfare, behaviour and lifestyle are important factors that may explain this relationship between socio-economic status and oral disease [5]. Individual and structural factors on a societal level both contribute to the oral health and disease status [7].

However, during the past decades different strategies have decreased the burden of oral disease. These strategies are both at individual and group/community level, such as different preventive measures; e.g. fluoride regimens [8], and increased access to dental care, resulting in improved dental visiting habits [1,5,6,9,10].

Consequently, oral health status has improved during the past 40 years in Sweden and other industrial countries, as assessed by the number of remaining teeth [9-15]. For example, it was stated in a review article by Muller et al. [15], that the number of edentulous individuals is falling in Europe, but also that there is a lack of population studies concerning edentulism and tooth loss in many countries. The authors also found that there are still large geographical differences in oral health status, according to the number of remaining teeth. These differences in oral health status also remain within the same country and between groups with different social backgrounds. Thus, it seems that the inequality in oral health status due to socio-economic and lifestyle factors prevails, even though the gradient has decreased in some countries [5,6,16-18].

Some Swedish studies report on the prevalence of edentulism over time. Thus, Ahlqwist et al. [11] have shown that edentulism among 50-year-old women has decreased significantly from 18% to 1% during a 24-year period. In their Jönköping-study, Hugoson et al. [10] revealed a significant drop in the prevalence of edentulism among 40 to 70 years old men and women during a 30-year period from 16%, 12%, 8% and 1% for the examination years 1973, 1983, 1993 and 2003, respectively. A similar trend has been shown in these studies with regard to number of teeth, with more remaining teeth the later the examination year, irrespective of age group [10,11].

In this paper, secular trends in oral health status regarding number of teeth over time are analysed. This was possible since the paper is based on the Population Study of Women in Gothenburg, Sweden, that was initiated in 1968. Hence, the specific aims of this investigation were to analyse secular trends in number of teeth and also to describe the differences in socio-economic status, among 38- and 50-year-old women, over a 36-year period.

## Methods

### Study population

The Population Study of Women in Gothenburg, Sweden (PSWG), was initiated in 1968/69 as a combined medical and dental health examination. Altogether, 1622 women born on specific days evenly divisible by six and living in Gothenburg were randomly selected, using a systematic sampling procedure, from the Revenue Office Register [19]. At the start of the study, the women were 38, 46, 50, 54 and 60 years of age. Altogether, 1417 women participated in the dental part of the study, which corresponds to a participation rate of 87.4% [13,19]. To invite the participants to the research unit, where all the health-examinations were performed, the women received a letter and subsequently a phone call. The high participation rate together with the sampling method may indicate that the sample was representative of women in the different age groups. However, caution should be taken into account when generalizing the results due to the characteristics of the non-participants.

The study was then performed with the same design in 12-year intervals, i.e. 1980/81, 1992/93 and 2004/05. In the dental part of these three last studies, the total number of women participating in each survey year were 1198, 994 and 500 (2004/05 only 38- and 50-year-old women were included) respectively. In all subsequent surveys new groups of 38-year-old women as well as new 50-year-olds were invited with the same inclusion criteria as the previous examination to ensure representativeness in all examinations (Table 1). In the last study women aged 50, who had participated in earlier studies in the PSWG, were invited even if they might have moved outside Gothenburg. Detailed information on the sampling procedure has been published previously [19-22].

**Table 1:** Number of women aged 38 and 50 years who participated in the dental examinations of the Population Study of Women in Gothenburg, and mean and median number of teeth and standard deviation (SD) in the studies in 1968/69, 1980/81, 1992/93 and 2004/05 *

		**1968/69**	**1980/81**	**1992/93**	**2004/05**
**Age 38**	Participants	356	109	67	207
	Participation rate	87.5%	75.2%	72.8%	59.5%
	Mean number of teeth	22.2	24.9	28.1	29.0
	Median	24.0	27.0	28.0	29.0
	SD	7.0	5.9	2.2	2.5
**Age 50**	Participants	390	323	98	293
	Participation rate	89.4%	76.4%	77.2%	57.7%
	Mean number of teeth	14.6	20.9	23.9	27.3
	Median	17.0	23.0	26.0	28.0
	SD	9.6	7.2	6.2	3.3

*Statistically significant differences (p < 0.05) concerning mean number of remaining teeth between all examinations and age groups except between 1992/93 and 2004/05 for the 38-year-olds.

The analyses of variance method was applied including a post-hoc analysis (LSD).

This study concerns the comparable age groups of women, 38 and 50 years old, in Gothenburg from the study in 1968/69 to 2004/05. The number of participants and participation rate in these age groups in the four different studies is given in Table 1.

### Non-participation analysis

The representativeness of the study groups has been revealed at each survey year by performing non-participation analyses including for example information concerning mortality, socio-economic status, number of teeth and, smoking habits. This information was obtained by telephone or mail and, from inpatient and outpatient records, as well as from the local fiscal authority [11,20,22].

In 1968/69 single women were over-represented among the non-participants. In the follow-up studies in 1980/81 and 1992/93 a higher proportion of the non-participants was edentulous and among the dentate individuals the non-participants had significantly fewer teeth left [11,20,23]. A higher proportion of the non-participating women was also smokers but showed no significant differences concerning socio-economic status. In 2004/05 the non-participants had a lower income and a higher proportion was immigrants [22].

### Study methods

The participants passed a series of examination stages, which included a dental radiographic examination (panoramic radiograph (OP)). The women answered questionnaires concerning socio-economic status as well as dental health and dental behaviour. In 1992/93 and 2004/05, a clinical examination of the dentition was also included. The number of teeth was assessed from the panoramic radiographs.

Marital status was given as not living together (i.e. single living, unmarried, divorced, widowed or married but not living together), or living together (i.e. cohabiting, married or in partnership).

Social class was divided into three categories. In the studies in 1968/69 and 1980/81, the married women reported their husband's occupation, but if not married, they reported their own occupation. In 1992/93 and 2004/05, the women's own occupation was reported in the first place. This information was transformed according to Carlson’s standard occupation grouping system [24]: low social group (skilled and unskilled workers), medium social group (small-scale employers, lower rank officials, foremen) and high social group (large-scale employers and high or intermediate rank officials).

Educational levels were based on years of school attendance and reported as: low (1–9 years), medium (10–12 years), and high level of education (≥13 years).

Income was measured in thousands of Swedish kronor (SEK) per year. The value in 1968/69 was recalculated according to the Consumer Price Index [25] to be comparable to the value in 2004/05 (the value in 2004/05 was 7.4 times higher than in 1968/69). However, information about income was not available for the study in 1980/81 and in 1992/93.

### Ethical approval

The Regional Ethical Review Board in Sweden approved the studies. Participation in the studies was voluntary, and all participants provided their written informed consent. To provide as much confidentiality as possible, we used anonymous patient-characteristic forms and anonymous questionnaires for data collection.

### Statistical analysis

The statistical analyses consisted of descriptive statistics and inference testing using the t-test, the Mann–Whitney test, the chi-square test, the one-way analysis of variance including a post-hoc test (LSD) and Spearman’s correlation analysis using SPSS 17.0. The chosen level of significance was p < 0.05.

Multivariate analyses were performed with logistic regression using different categorizations of the outcome variable, number of teeth. Thus the number of teeth was categorized into a binary variable; 1+ vs 0 teeth (Model I), 11+ vs 0–10 teeth (Model II), 21+ vs 0–20 teeth (Model III), and 25+ vs 0–24 teeth (Model IV). The examination years were included in the models for a time series analysis (linear trend over time). This covariate was used as a continuous variable with 1968 = 1 up to 2004 = 4 indicating a change in the odds ratio per twelve years. The other independent variables included were age, social group and marital status. Possible interaction terms between the linear trend over time and other variables were not significant (data not shown).

**Table 2:** Percentage number of women aged 38 and 50 years in the studies from 1968/69 to 2004/05 according to marital status, social class and education

		**1968/69**	**1980/81**	**1992/93**	**2004/05**
**Age 38**	Living together	82.6	64.8a	62.1a	44.3
	Single living	17.4	35.2a	37.9a	55.7
	High social group	10.5bc	15.3b	10.6c	24.6
	Medium social group	51.7bc	39.8b	59.1c	48.3
	Low social group	37.8bc	44.9b	30.3c	27.1
	High education	1.4	10.7	30.5	56.2
	Medium education	11.6	18.0	25.4	40.9
	Low education	87.0	71.3	44.1	3.0
**Age 50**	Living together	77.9b	73.5ab	65.3a	53.1
	Single living	22.1b	26.5ab	34.7a	46.9
	High social group	12.7b	10.9ab	7.3a	19.2
	Medium social group	43.2b	49.4ab	59.4a	50.7
	Low social group	44.0b	39.7ab	33.3a	30.1
	High education	1.3b	1.4b	9.8	50.7
	Medium education	13.1b	14.2b	28.3	37.6
	Low education	85.6b	84.4b	62.0	11.7

Significance (p < 0.05) in sub-analyses between the study years in all groups except for:

a = no significant difference between the study 1980/81 and 1992/93 in sub-analyses.

b = no significant difference between the study 1968/69 and 1980/81 in sub-analyses.

c = no significant difference between the study 1968/69 and 1992/93 in sub-analyses.

## Results

### Number of teeth

The mean number of remaining teeth increased significantly for both 38- and 50-year-old women during this 36-year span (Table 1). The later examination year, the more teeth were left. Edentulism (data not shown in Table 1) significantly decreased over time, especially among the 50-year-olds, where as many as 18.2% were edentulous in 1968/69, and only 0.3% in 2004/05 (p < 0.001). Among the 38-year-olds, 3.9% were edentulous in 1968/69 and none in 2004/05. All the women, except one, in this later cohort had 21 or more remaining teeth in 2004/05.

### Marital status

The number of women living together with a partner decreased during the time span of 36 years (Table 2). Among the women in both age groups, there was an increase in the number of women living alone in 2004/05 compared to 1968/69 from around 20% to around 50% (Table 2).

**Table 3:** Mean number of teeth and standard deviation (SD) according to marital status, social group and education among women aged 38 and 50 years, in the studies from 1968/69 to 2004/05

		**1968/69**		**1980/81**		**1992/93**		**2004/05**	
		**Mean**	**(SD)**	**Mean**	**(SD)**	**Mean**	**(SD)**	**Mean**	**(SD)**
**Age 38**	Living together	22.5	(6.8)	24.9	(6.2)	28.4	(2.1)	28.6	(2.7)
	Single living	20.6	(7.8)	25.0	(5.4)	27.6	(2.2)	29.1	(2.3)
	High social group	25.8a	(3.2)	27.4b	(2.0)	27.1	(2.6)	28.8	(2.2)
	Medium social group	22.7a	(6.5)	25.5	(5.5)	28.2	(2.0)	29.1	(2.7)
	Low social group	20.5a	(8.1)	24.0b	(6.4)	28.1	(2.4)	28.8	(2.5)
	High education	27.4	(2.4)	28.0c	(1.6)	28.2	(2.1)	29.2	(2.1)
	Medium education	24.7b	(4.4)	27.1b	(2.3)	28.1	(2.5)	28.7	(2.9)
	Low education	21.8b	(7.3)	23.9a	(6.6)	27.8	(2.2)	27.3	(4.2)
**Age 50**	Living together	15.1*	(9.4)	21.8*	(6.4)	23.7	(6.3)	27.7*	(2.4)
	Single living	12.7*	(9.9)	18.0*	(8.7)	24.1	(6.2)	27.0*	(3.8)
	High social group	21.4a	(6.5)	23.7b	(4.9)	27.6b	(0.5)	27.9b	(2.2)
	Medium social group	16.4a	(8.8)	21.3c	(7.0)	25.0c	(5.1)	27.7c	(2.5)
	Low social group	11.0a	(9.4)	19.6bc	(7.8)	21.1bc	(7.6)	26.1bc	(4.6)
	High education	21.6	(10.0)	25.3	(4.3)	28.1c	(1.5)	28.2a	(2.2)
	Medium education	20.5b	(8.3)	22.6	(6.1)	25.9b	(2.5)	26.9a	(3.0)
	Low education	13.6b	(9.4)	20.6	(7.4)	22.8a	(6.9)	25.1a	(5.3)

Marital status and number of teeth: * = Significance (p < 0.05) between the marital status groups.

Social group and number of teeth:

a = significance (p < 0.05) between all social groups.

b = significance (p < 0.05) between high and low social groups.

c = significance (p < 0.05) between medium and low social groups.

Educational level and number of teeth:

a = significance (p < 0.05) between all education groups.

b = significance (p < 0.05) between low and medium education groups.

c = significance (p < 0.05) between low and high education groups.

The analyses of variance method was applied including a post-hoc analysis (LSD).

The 50-year-old women living together with a partner had significantly more remaining teeth than those who lived alone (Table 3), except in 1992/93. However for the 38-year-olds, there was no significant relationship between the number of remaining teeth and marital status (Table 3).

**Table 4:** The multivariate logistic regression with number of teeth as the dependent variable and, linear trend over time (study year), age, marital status and social group as the independent variables

**Independent variables**	**P-value**	**Odds ratio**	**95% confidence interval**
**Dependent variable: Number of teeth 1+ vs 0 teeth (Model I)**			
Linear trend over time	<0.001	0.20	0.13-0.31
38 years old (reference)			
50 years old	<0.001	4.79	2.73-8.43
Living together (reference)			
Single living	0.028	1.74	1.06-2.84
High social group (reference)			
Medium social group	0.051	7.42	0.99-55.45
Low social group	0.002	24.72	3.38-180.96
**Dependent variable: Number of teeth 11+ vs 0–10 teeth (Model II)**			
Linear trend over time	<0.001	0.21	0.15-0.28
38 years old (reference)			
50 years old	<0.001	5.09	3.45-7.50
Living together (reference)			
Single living	0.004	1.74	1.20-2.52
High social group (reference)			
Medium social group	0.001	4.86	1.90-12.43
Low social group	<0.001	11.57	4.58-29.35
**Dependent variable: Number of teeth 21+ vs 0–20 teeth (Model III)**			
Linear trend over time	<0.001	0.25	0.21-0.30
38 years old (reference)			
50 years old	<0.001	4.94	3.72-6.55
Living together (reference)			
Single living	0.001	1.63	1.21-2.20
High social group (reference)			
Medium social group	<0.001	2.92	1.78-4.80
Low social group	<0.001	5.61	3.41-9.23
**Dependent variable: Number of teeth 25+ vs 0–24 teeth (Model IV)**			
Linear trend over time	<0.001	0.30	0.26-0.34
38 years old (reference)			
50 years old	<0.001	4.39	3.42-5.63
Living together (reference)			
Single living	0.005	1.48	1.13-1.93
High social group (reference)			
Medium social group	<0.001	2.13	1.47-3.10
Low social group	<0.001	4.13	2.81-6.08

### Social group

For both 38- and 50-year-olds there has been a significant shift concerning the social class belonging. More women were categorized in a higher social group in 2004/05 than in 1968/69 (Table 2).

Both age groups had a significant relationship between fewer teeth and a low social group. For the 50-year-olds this was irrespective of examination year, and for the 38-year-olds this was seen only in the first (1968/69) and second (1981/82) study (Table 3).

### Educational level

During the 36 years, the educational level of the women changed significantly. Among women aged 38 years in 1968/69, a big majority had only attended elementary school (low education) (Table 2). In 2004/05 the situation was the reverse, with only 3% in the group with only elementary school. Among the 50-year-olds the educational level also increased from a big majority in the low educational group in 1968/69 to only 11% in the same group in 2004/05.

In 1968/69 and 1980/81 the 38-year-olds with low education had significantly fewer remaining teeth left (Table 3). In the two later studies (1992/93 and 2004/05) the mean number of teeth was about the same, regardless of educational level.

The 50-year-old women with a low educational level had significantly fewer remaining teeth than those with a higher educational level in all studies except for 1980/81 (Table 3).

### Income

Most women had their own income in 2004/05 and they also had a significantly higher income than in the study in 1968/69, calculated according to the Consumer Price Index (25). Only 5.7% of the women aged 38 and 50 years stated that they had no income at all in 2004/05, compared to 33.7% in 1968/69. Even though the husbands of the participating women had a higher mean income in the study in 1968/69 than in 2004/05, the income for the total household (for a couple) increased for both age groups from 1968/69 to 2004/05.

The correlation coefficients were low when analyses were made between the mean number of teeth and income for the households. For 38-year-old women r = 0.15 (p = 0.01) in 1968/69 and r = 0.02 (NS) in 2004/05 and, for the 50-year-olds r = 0.32 (p < 0.001) in 1968/69 and r = 0.17 (p = 0.003) in 2004/05, which means that there is only a slight indication that the higher the income, the more remaining teeth.

### Multivariate analyses

Age, marital status, social group and examination year had a clear impact on the risk of having fewer teeth (Table 4). Thus, women aged 50 years had about 4 to 5 times higher odds ratio than the 38-year-olds to have fewer teeth. Single living women had also a significantly higher odds ratio of having fewer teeth than women living together with a partner (odds ratio of 1.4 to 1.7). Low social group belonging had the highest significant impact on the odds of having fewer teeth with odds ratio 4.1-24.7. The impact of social group on the number of teeth decreased in the models from I to IV, as shown by the absolute odds ratios for each model. Thus low social group resulted in higher risk of having fewer teeth.

It was also shown that the time for examination had a protective effect on the number of teeth i.e. the later the women had their survey, the less risk of belonging to the categories with fewer teeth.

## Discussion

The main findings from this study were a significant increase in the mean number of teeth among women both aged 38 and 50 years and a substantial decrease in edentulism, over a study period of 36 years. Moreover, we found a relationship between fewer teeth and lower social group and, also between fewer teeth and low education, among the 50-year-old women, irrespective of examination year. However, for the 38-year-olds these results only adhere to the first two surveys. Marital status just affected the number of remaining teeth for the 50-year-olds.

However the multivariate analysis, including a time trend analysis, indicated important findings. Irrespective of examination year and categorizations of the variable “teeth” according to the models I-IV, we found that social group, marital status and age group were significant predictors of oral health status according to number of teeth. In addition, the analysis also showed that the women in the later surveys had a lower risk to belong to the categories with fewer teeth.

The study design had some weaknesses, one being that the sample sizes were moderately large, especially the 1992/93 cohorts (see Table 1). The measurement methods have been the same, however the variable educational level has changed over time, which altered the number of years in the mandatory elementary school. Hence, we categorized the educational level according to the number of years in the school system, thereby minimizing misclassification. However, due to the self reported character of the variables, caution must be taken with respect to possible bias since we do not know if the women have changed their interpretations of questionnaires and self-report over time. Furthermore, the samples only included women. Another weakness of this study was the declining participation rate over the years. In the first study it was as high as 90%, declining to around 60% in 2004/05. We have made several non-participation analyses, which showed that the non-participants had a smaller number of remaining teeth (1980/81 and 1992/93) than the participants [20] and had a lower income and were more often immigrants in 2004/05 [22]. These facts may influence the result to some extent and the improvement in oral health status may be somewhat overestimated.

The strengths of the study were the randomly selected individuals and the repeated cross-sectional design over the long period, which may be an important key to elucidate significant changes in health and socio-economic patterns in a Swedish urban population.

It is shown in studies that it has been a substantial improvement in oral health status over the past decades with regard to the number of remaining teeth [5,10,26]. However, there is still a need to further explore the relationship between oral health status and socio-economic status, as the literature still reveals inequality in oral health related to social group [16-18,26,27].

The increase in the number of remaining teeth over the study period was remarkable among both age groups. For example, in 1968/69 the mean number of teeth was 14.6 compared to 27.3 in 2004/05 for the 50-year-old women. However, the number of remaining teeth was still significantly related to social group in all the studies, but also to educational level except in 1980/81. For the 38-year-old women the mean number of teeth was 29.0 in 2004/05 compared to 22.2 in 1968/69. In this group, which especially in the latest study seemed to have lost very few teeth, the significant differences in number of teeth and social belonging, as well as to educational level disappeared in the two latest studies.

Similar findings with decreased tooth mortality have been reported from other cohort comparison studies in other industrialized countries. Holst [5] concluded that the proportion of individuals in Norway aged 35 to 59 who reported more than 20 remaining teeth increased significantly over time, albeit with smaller differences with respect to socio-economic position. Another study performed in 1990 and 2002, in the Northern part of Sweden, showed that tooth mortality decreased significantly but was still related to social factors such as level of education, except for the youngest age group of 35-year-olds [26] which is in accordance with the result from our youngest age group.

When we compared women with regard to marital status, we noted a secular trend of a remarkable increase towards single-living women over the 36-year period in both age groups. The present study showed that the 50-year-old women living together with a partner had significantly more teeth left than women living alone, which has been shown in all examinations except in 1992/93. No such relationship was found for the 38-year-olds. However, “living alone” was a risk factor both for a lower number of teeth and edentulism, independent of age, in the multivariate study. This influence of marital status on the number of remaining teeth has also been found in other studies, where married women have a larger number of remaining teeth [28,29] and “not married” has been shown to be a risk factor for tooth loss in both older men and women [30].

As we have seen during a time span of 36 years, the society in which we live certainly changes. Thus, comparisons between different cohorts of individuals over a long period should probably include different aspects, over and above revealed differences in the number of remaining teeth. Such effects may be due to cohort, age and/or period changes. Ahacic et al. [31] investigated such effects over a period of 34 years. Their findings indicated small changes in edentulism longitudinally within cohorts, while large differences were shown between cohorts. Parallel results were indicated in our study, showing a secular trend with significantly more remaining teeth over the period 1968/69 to 2004/05; however, no longitudinal analysis was performed due to the study design. A certain age effect has been revealed, especially in the first two surveys.

## Conclusions

This study of secular trends, with a follow-up period of 36 years, showed that oral health according to number of remaining teeth improved in comparable age groups. Fewer individuals (i) had lost all their teeth and (ii) dentate individuals had more remaining teeth, even though differences due to social group, level of education, marital status and, age were still found.

A time trend analysis indicated that individuals in the later examination years had fewer teeth lost, irrespective of the other independent variables such as age, marital status and, social group.

## Competing interests

The authors declare that they have no competing interests.

## Authors’ contributions

AW and MH performed the analyses. AW, MH and MA did the interpretation of the results and wrote the manuscript. CB and MH designed the study. AW, MA, US and MH collected the data. AW, MA, US, MH and CB drafted the manuscript. All authors read and approved the final manuscript.

## Pre-publication history

The pre-publication history for this paper can be accessed here:

http://www.biomedcentral.com/1472-6831/13/63/prepub
